# Effect of Structured Exercise for the Rehabilitation of Patients With Chronic Parkinson’s Disease

**DOI:** 10.7759/cureus.110144

**Published:** 2026-06-02

**Authors:** Mukta T Killedar, Suraj B Kanase

**Affiliations:** 1 Department of Neurosciences, Krishna College of Physiotherapy, Krishna Vishwa Vidyapeeth (Deemed to be University), Karad, IND

**Keywords:** berg balance scale, functional mobility, neurorehabilitation, parkinson’s disease, physiotherapy, timed up and go test

## Abstract

Introduction

Parkinson’s disease is a progressive neurological condition that gradually affects movement, balance, and independence in daily activities. While medication remains the primary form of treatment, it often does not fully address functional limitations or postural instability. In recent years, structured exercise has gained importance as a supportive rehabilitation approach due to its potential to enhance motor function and promote neural adaptability. This study was designed to assess the effect of a structured exercise program on functional mobility, balance, and disease severity in individuals with chronic Parkinson’s disease.

Methods

A pre-post experimental study was conducted in the Neurosciences Outpatient Department (OPD) at Krishna College of Physiotherapy, Karad. A total of 20 individuals diagnosed with chronic Parkinson’s disease, aged between 50 and 70 years and classified within Hoehn and Yahr stages II-V, were recruited through consecutive sampling. The participants underwent a structured exercise program for six weeks, five days per week, with each session lasting 45 minutes. Outcomes were assessed using the Timed Up and Go (TUG) test, Berg Balance Scale (BBS), and Hoehn and Yahr staging before and after the intervention. Data were analyzed using paired t-test and Wilcoxon signed-rank test, with statistical significance set at p < 0.05.

Results

Following the intervention, significant improvements were observed across all measured outcomes. Functional mobility improved, with TUG scores decreasing from 18.6 ± 3.2 seconds to 14.2 ± 2.8 seconds (p < 0.001; Cohen’s d = 1.75). Balance showed notable enhancement, with BBS scores increasing from 34.5 ± 5.1 to 42.8 ± 4.7 (p < 0.001; d = 1.83). Disease severity reduced significantly, with Hoehn and Yahr stages decreasing from 3 (IQR: 3-4) to 2 (IQR: 2-3) (p < 0.001).

Conclusion

A six-week structured exercise program resulted in meaningful improvements in mobility, balance, and disease severity in individuals with chronic Parkinson’s disease. These findings support the inclusion of structured exercise as an effective, low-cost, and clinically valuable component of Parkinson’s disease rehabilitation to improve functional outcomes and the overall quality of life.

## Introduction

Parkinson’s disease is a chronic progressive neurodegenerative disorder that primarily affects movement and functional performance. It is commonly characterized by motor symptoms such as bradykinesia, muscular rigidity, resting tremor, and postural instability, along with non-motor manifestations including cognitive impairment, depression, and autonomic dysfunction [1]. The disorder primarily results from the degeneration of dopaminergic neurons within the substantia nigra, leading to impaired basal ganglia function and the progressive deterioration of motor control [2].

Multiple pathological mechanisms contribute to the development and progression of Parkinson’s disease, including oxidative stress, mitochondrial dysfunction, neuroinflammation, and the abnormal accumulation of alpha-synuclein protein [2]. These pathological changes progressively impair motor coordination, balance, gait, and functional independence. In recent years, neuroplasticity has gained significant importance in Parkinson’s disease rehabilitation because the nervous system retains the ability to reorganize and adapt in response to repetitive therapeutic training. Exercise-based rehabilitation has been associated with improved cortical activation, enhanced synaptic efficiency, and better motor learning, thereby contributing to functional recovery in affected individuals [3].

The prevalence of Parkinson’s disease has increased considerably worldwide over recent decades, resulting in a growing healthcare and socioeconomic burden [4]. Disease severity and progression are commonly classified using the modified Hoehn and Yahr staging system, which categorizes patients from early unilateral involvement to severe disability with impaired independent mobility in advanced stages [5]. Early-onset forms of Parkinsonism may additionally present with distinct genetic and clinical characteristics [6]. As the disease progresses, individuals frequently experience deterioration in balance, gait, and mobility, significantly affecting the overall quality of life and community participation.

Pharmacological management remains the primary approach for the symptomatic treatment of Parkinson’s disease, with levodopa and dopamine agonists commonly prescribed to improve motor symptoms [1]. However, pharmacological treatment alone is often insufficient to address impairments related to postural control, balance, mobility, and functional performance. Long-term medication use may additionally result in complications such as dyskinesias and motor fluctuations. Therefore, physiotherapy and neurorehabilitation have become essential components of comprehensive Parkinson’s disease management [7].

Exercise has emerged as an important adjunct therapeutic strategy because of its beneficial effects on neuroplasticity and physical function. Regular structured exercise may improve motor relearning, enhance neural connectivity, and support the preservation of functional abilities. Consequently, rehabilitation interventions are increasingly being incorporated into long-term management programs for individuals with Parkinson’s disease.

Previous studies have demonstrated the beneficial effects of various exercise-based rehabilitation approaches in Parkinson’s disease. Aerobic and treadmill-based exercise interventions have shown positive effects on gait performance, walking endurance, and mobility outcomes [8,9]. Resistance training programs have additionally been associated with improvements in muscle strength, physical performance, and motor function [10,11]. Similarly, task-specific, cue-based, and adaptive movement training approaches have demonstrated beneficial effects on motor coordination, bradykinesia, and functional mobility [12,13]. Collectively, these findings support the role of structured exercise interventions in improving functional performance in individuals with Parkinson’s disease.

Alternative rehabilitation approaches such as Pilates, hydrotherapy, and tandem cycling have also demonstrated clinically beneficial outcomes in Parkinson’s disease rehabilitation. These interventions have been associated with improvements in balance, endurance, postural stability, and overall physical performance [14-16]. Such findings indicate that rehabilitation programs incorporating different modes of exercise may help address the multidimensional impairments observed in chronic Parkinson’s disease.

Appropriate outcome assessment is essential in Parkinson’s disease rehabilitation research to evaluate the effectiveness of therapeutic interventions. Functional mobility can be assessed using the Timed Up and Go (TUG) test [17], while balance and postural stability may be evaluated using the Berg Balance Scale (BBS) [18]. Additionally, the Hoehn and Yahr staging system is widely used to classify disease severity and monitor disease progression in individuals with Parkinson’s disease [5]. Functional mobility tests have also been shown to be responsive to physical therapy interventions in Parkinson’s disease rehabilitation [19]. The selected outcome measures in the present study were chosen to assess clinically relevant domains commonly affected in chronic Parkinson’s disease, particularly mobility, balance, and disease severity.

Although previous studies have demonstrated the benefits of physiotherapy interventions in Parkinson’s disease, many investigations have primarily focused on early-stage or mixed-stage populations. Limited evidence is available regarding the effectiveness of structured exercise interventions specifically in individuals with chronic Parkinson’s disease across different Hoehn and Yahr stages. For the purpose of the present study, individuals with chronic Parkinson’s disease were operationally defined as patients with a clinically confirmed diagnosis of Parkinson’s disease presenting with persistent and progressive motor impairments, functional limitations, and the need for ongoing physiotherapy rehabilitation. Since chronic Parkinson’s disease is frequently associated with progressive decline in mobility, there is a need for rehabilitation approaches that can help maintain physical function and improve the quality of movement in this population.

Unlike many previous studies that primarily focused on isolated exercise interventions or limited stages of Parkinson’s disease, the present study investigated a structured stage-based physiotherapy rehabilitation program tailored according to disease severity across different functional stages of chronic Parkinson’s disease, thereby providing a more comprehensive and clinically applicable rehabilitation approach. Therefore, the present study aimed to evaluate the effects of this structured rehabilitation program on functional mobility, balance, and disease severity in individuals with chronic Parkinson’s disease across different stages of the modified Hoehn and Yahr scale.

## Materials and methods

This single-group pretest-posttest quasi-experimental study was conducted from November 5, 2025, to March 31, 2026, at the Neurosciences Outpatient Department (OPD) at Krishna College of Physiotherapy, Karad. The study aimed to evaluate the effectiveness of a structured physiotherapy exercise program on functional mobility, balance, and disease severity in individuals with chronic Parkinson’s disease. Ethical approval was obtained from the Institutional Ethics Committee of Krishna Vishwa Vidyapeeth (Deemed to be University) (approval number: KVV/IEC/10/2025) prior to commencement of the study.

The participants were recruited using a consecutive sampling method from the Neurosciences Outpatient Department. The study included 20 individuals diagnosed with chronic Parkinson’s disease, comprising 10 men and 10 women aged 50-70 years. All participants underwent screening based on predefined eligibility criteria before enrolment. Considering the exploratory nature of the single-group pretest-posttest quasi-experimental study design and the feasibility of participant recruitment during the study period, a formal a priori sample size calculation was not performed. Therefore, a feasibility-based convenience sample of eligible participants available during the study duration was included.

For the purpose of the present study, individuals with chronic Parkinson’s disease were operationally defined as patients with a clinically confirmed diagnosis of Parkinson’s disease presenting with persistent and progressive motor impairments, functional limitations, and the need for ongoing physiotherapy rehabilitation.

The participants with a confirmed diagnosis of chronic Parkinson’s disease classified within modified Hoehn and Yahr stages II-V were eligible for inclusion. Disease severity was classified using the modified Hoehn and Yahr staging system [5,20]. The scale categorizes Parkinson’s disease into five stages according to symptom progression and functional disability. Stage I represents unilateral involvement only, stage II indicates bilateral involvement without balance impairment, stage III denotes mild-to-moderate bilateral disease with postural instability while maintaining physical independence, stage IV represents severe disability with preserved ability to stand or walk independently, and stage V corresponds to advanced disease in which patients are wheelchair-bound or bedridden unless aided [5,20].

The participants commonly presented with motor impairments, including bradykinesia, rigidity, resting tremor, gait dysfunction, impaired balance, postural instability, coordination deficits, and the freezing of gait. Additional inclusion criteria included medical stability, ability to understand and follow simple verbal instructions, and the capacity to stand and ambulate independently or with assistance.

Individuals with severe cognitive impairment or dementia, neurological disorders other than Parkinson’s disease affecting mobility (such as stroke or multiple sclerosis), severe musculoskeletal conditions limiting balance or lower-limb function, recent fractures or orthopedic surgery within the preceding six months, severe visual or vestibular impairment affecting postural control, or medical conditions contraindicating moderate physical exercise were excluded.

Functional mobility was assessed using the Timed Up and Go (TUG) test [17]. The participants were instructed to rise from a standard chair, walk 3 meters, turn around, return to the chair, and sit down. The total completion time was recorded in seconds. Lower scores indicated better functional mobility, whereas higher scores reflected greater mobility limitation and increased fall risk [17].

Balance and postural control were evaluated using the Berg Balance Scale (BBS) [18]. The BBS consists of 14 functional balance tasks scored on a five-point ordinal scale, ranging from 0 to 4, with a maximum total score of 56. Higher scores indicate better balance performance and postural stability, whereas lower scores reflect greater balance impairment and increased fall risk [18].

Outcome assessments were completed at baseline and after the completion of the intervention period.

The intervention consisted of a structured physiotherapy rehabilitation program delivered over six weeks, five days per week, with each session lasting 45 minutes. The program targeted functional mobility, balance, postural stability, and functional movement control in individuals with chronic Parkinson’s disease. The intervention followed a stage-based rehabilitation approach, with exercise selection and progression guided by disease severity and clinical presentation. Exercise intensity was prescribed and progressed according to patient tolerance, clinical presentation, and rating of perceived exertion (RPE) levels to ensure safe and standardized exercise delivery. Progression criteria included the ability to perform prescribed exercises with proper movement quality, stable balance, minimal fatigue, and without adverse symptoms such as dizziness, excessive exhaustion, or the loss of postural control. Based on patient performance and clinical response, progression was achieved by gradually increasing repetitions, exercise duration, resistance, gait complexity, balance challenge, coordination demands, and task-specific functional activities. Participant adherence was monitored through attendance records maintained during each supervised rehabilitation session. All intervention sessions were conducted under the supervision of qualified physiotherapists to ensure patient safety, correct exercise performance, and the appropriate progression of the rehabilitation program. The detailed stage-specific exercise protocol is presented in Table 1.

**Table 1:** Structured Ability-Based Physiotherapy Rehabilitation Protocol Across Modified Hoehn and Yahr Stages in Individuals With Chronic Parkinson’s Disease ROM, range of motion; ADL, activities of daily living; RPE, rating of perceived exertion; reps, repetitions

Stage	Clinical Features	Structured Rehabilitation Interventions (Fully Detailed)
Stage 1	Unilateral symptoms; minimal functional limitation; mild motor impairment	Breathing exercises: diaphragmatic and thoracic expansion exercises in supine/sitting, 10-15 repetitions × 2-3 cycles to improve ventilation and relaxation
		Active ROM exercises: full active movements of upper- and lower-limb joints (10-15 reps/joint) to maintain mobility and motor control
		Flexibility training: sustained stretching of major muscle groups (20-30-second hold × 3-5 reps) to prevent early stiffness
		Strength training: body-weight or light resistance exercises (two sets × 10 reps; RPE: 11-13) based on patient tolerance
		Aerobic training: walking or cycling for 10-20 minutes at moderate intensity based on patient endurance
		Postural training: mirror and verbal feedback-based upright posture training to improve alignment and symmetry
Stage 1.5	Early axial involvement; mild functional limitations	Task-specific training: repetitive functional movements such as reaching, grasping, and stepping (10-15 reps per task) based on patient ability
		Trunk mobility exercises: controlled flexion, extension, and rotation (10-15 reps) to improve axial control
		Transfer training: sit to stand, bed mobility, and directional transfers (8-12 repetitions) adjusted according to patient independence level
		Relaxation training: breathing and muscle relaxation techniques (5-10 minutes) to reduce rigidity and improve movement quality
		Resistance training: low-load strengthening using bands or body weight (two sets × 10 reps), progressed as tolerated
		Movement retraining: external cueing strategies, including auditory and visual cueing, were incorporated during the intervention sessions to facilitate movement initiation and improve motor performance
Stage 2	Bilateral involvement; mild rigidity; mild functional limitation	Functional mobility training: repetitive practice of ADL-related tasks (walking, turning, and reaching) adjusted to patient performance level
		Gait training: overground walking (10-20 minutes) with focus on stride length and arm swing, progressed based on endurance and stability
		Active ROM exercises: full joint mobility exercises (10-15 reps/joint)
		Stretching exercises: hamstrings, calf, hip flexors, and shoulder muscles (20-30 seconds × 3-5 reps)
		Core strengthening: abdominal and paraspinal activation (two sets × 10 reps)
		Facial and speech exercises: oromotor and phonation training (5-10 minutes) as tolerated
		Postural correction: feedback-based alignment training using a mirror and tactile cues
		Balance training: static balance exercises introduced and progressed based on stability (5-15 minutes)
Stage 2.5	Mild balance impairment; good recovery on pull test	Balance training (static and dynamic): tandem stance, single-leg stance (as tolerated), and weight shifting (10-20 minutes), progressed based on safety and control
		Coordination training: marching, side stepping, backward walking, and figure-of-eight walking (10-15 minutes), adjusted to patient coordination level
		Strength training: lower- and upper-limb strengthening (two sets × 10 reps), progressed according to fatigue tolerance
		Proprioceptive training: joint position awareness and controlled movement correction based on patient feedback ability
		Trunk stabilization: core activation exercises progressed based on postural control
Stage 3	Moderate disease; postural instability; independent but fall risk	Balance training: challenging static and dynamic balance tasks (15-20 minutes) progressed according to stability and safety response
		Gait training with dual tasking: walking with cognitive or motor tasks (10-15 minutes) only if the patient can maintain safety
		Resistance training: moderate intensity strengthening (2-3 sets × 10-12 reps) adjusted to fatigue and functional capacity
		Core stabilization (Pilates-based): controlled breathing and trunk control exercises progressed based on motor control
		Functional strengthening: task-based activities (squatting and lifting) modified according to ability
		Coordination training: rhythmic and patterned movement training based on performance capacity
Stage 4	Severe disability; ambulatory with assistance	Assisted gait training: walking with support for 5-10 minutes based on safety and fatigue
		Transfer training: bed, chair, and toilet transfers, 8-10 repetitions with graded assistance
		Trunk mobility exercises: assisted trunk movements (flexion, extension, and rotation), 10 repetitions within a safe range
		Strength training: low-intensity, assisted or gravity-eliminated exercises, two sets × 8-10 repetitions
		Respiratory exercises: deep breathing and thoracic expansion, 10 repetitions × three sets
		Swallowing training: oromotor exercises based on tolerance
		Functional task training: assisted ADL practice to maintain the independence level
Stage 5	Wheelchair-bound or bedridden	Bed mobility training: rolling, repositioning, and supine to sit, 5-10 repetitions based on ability
		Passive ROM exercises: gentle passive movements for all joints, 10-15 repetitions per joint to prevent contractures
		Positioning and pressure care: Regular repositioning to prevent pressure sores
		Caregiver-assisted movements: assisted limb mobilization to maintain circulation and prevent stiffness
		Respiratory exercises: deep breathing and chest expansion, 10 repetitions × three sets
		Seated balance training: only if tolerated safely in the sitting position

Data analysis was performed using IBM SPSS Statistics version 31.0 (IBM Corp., Armonk, NY). Continuous variables were summarized as mean ± standard deviation, whereas categorical variables were expressed as frequency and percentage. Data normality was assessed using the Shapiro-Wilk test. Within-group comparisons for TUG and BBS outcomes were analyzed using the paired t-test. Because Hoehn and Yahr staging represents ordinal data, changes in disease severity were analyzed using the Wilcoxon signed-rank test. A p-value of <0.05 was considered statistically significant. Effect size was calculated using Cohen’s d and interpreted as small (0.2), moderate (0.5), and large (≥0.8).

## Results

The study included a total of 20 participants, with an equal distribution of men and women (50% each). The mean age of the participants was 60.4 ± 5.8 years, with an age range of 50-70 years, indicating a relatively homogenous middle-aged to older adult study population. The demographic characteristics of the study participants with chronic Parkinson’s disease are presented in Table 2.

**Table 2:** Demographic Characteristics of the Participants (n = 20) The age range of the study participants was 50-70 years SD, standard deviation; n, number of participants

Variable	Value
Age (years), mean ± SD	60.4 ± 5.8
Gender, male	10 (50%)
Gender, female	10 (50%)

No participants were classified within stage 1 or stage 1.5 of the modified Hoehn and Yahr staging system. Among the study population, 20% of the participants were classified within stage 2, 30% within stage 2.5, 25% within stage 3, 15% within stage 4, and 10% within stage 5, indicating the predominance of mild-to-moderate disease severity among the participants, as shown in Table 3.

**Table 3:** Distribution of Participants According to Modified Hoehn and Yahr Stages (n = 20)

Modified Hoehn and Yahr Stage	Number of Participants (n)	Percentage (%)
Stage 1	0	0%
Stage 1.5	0	0%
Stage 2	4	20%
Stage 2.5	6	30%
Stage 3	5	25%
Stage 4	3	15%
Stage 5	2	10%

A statistically significant improvement was observed across all functional outcome measures following the intervention. Functional mobility, assessed using the Timed Up and Go (TUG) test, showed a significant reduction in completion time from 18.6 ± 3.2 seconds at baseline to 14.2 ± 2.8 seconds post-intervention (t = 7.84, degrees of freedom {df} = 19, p < 0.001, and Cohen’s d = 1.75), indicating improved mobility and the reduced risk of falls as shown in Table 4.

**Table 4:** Pre-Post Comparison of Functional Mobility Assessed Using Timed Up and Go (TUG) Test in Chronic Parkinson’s Disease Comparison of pre-intervention with post-intervention Timed Up and Go test using paired t-test df, degrees of freedom; SD, standard deviation

Outcome Measure	Pre-intervention (Mean ± SD)	Post-intervention (Mean ± SD)	T value	df	P-value	Cohen’s d
TUG (seconds)	18.6 ± 3.2	14.2 ± 2.8	7.84	19	<0.001	1.75

Balance performance, measured using the Berg Balance Scale (BBS), improved significantly from 34.5 ± 5.1 to 42.8 ± 4.7 (t = 8.21, df = 19, p < 0.001, and Cohen’s d = 1.83), reflecting enhanced postural stability as shown in Table 5.

**Table 5:** Pre-Post Comparison of Balance Assessed Using Berg Balance Scale (BBS) in Chronic Parkinson’s Disease Comparison of pre-intervention with post-intervention balance outcome using paired t-test df, degrees of freedom; SD, standard deviation

Outcome Measure	Pre-intervention (Mean ± SD)	Post-intervention (Mean ± SD)	T value	df	P-value	Cohen’s d
BBS	34.5 ± 5.1	42.8 ± 4.7	8.21	19	<0.001	1.83

Disease severity, evaluated using the Hoehn and Yahr scale, showed a statistically significant improvement, with median stage reducing from 3 (IQR: 3-4) at baseline to 2 (IQR: 2-3) post-intervention (Z = -3.62; p < 0.001). The effect size was not applicable for this ordinal variable as shown in Table 6.

**Table 6:** Pre-Post Comparison of Disease Severity Assessed Using the Modified Hoehn and Yahr Scale in Chronic Parkinson’s Disease Comparison of pre-intervention with post-intervention modified Hoehn and Yahr scale scores using the Wilcoxon signed-rank test df, degrees of freedom; SD, standard deviation

Outcome Measure	Pre-intervention (Mean ± SD)	Post-intervention (Mean ± SD)	Z value	df	P-value	Cohen’s d
Hoehn and Yahr	3 (3-4)	2 (2-3)	-3.62	-	<0.001	-

Overall, the structured exercise program resulted in statistically significant and clinically meaningful improvements in functional mobility and balance, along with a reduction in disease severity. The magnitude of improvement across all outcome measures indicates a large clinical effect of the intervention in individuals with chronic Parkinson’s disease.

## Discussion

The present study investigated the effectiveness of a structured, ability-based physiotherapy program on functional mobility, balance, and disease severity in individuals with chronic Parkinson’s disease. Significant improvements were observed in TUG, BBS, and modified Hoehn and Yahr scores following intervention, suggesting that a multi-component rehabilitation approach can produce meaningful functional benefits in this population.

Improvement in functional mobility, demonstrated by reduced Timed Up and Go (TUG) scores, indicates enhanced gait performance and motor control following the intervention. Previous evidence suggests that exercise-based rehabilitation promotes neuroplasticity by improving synaptic efficiency and motor learning mechanisms [3]. Treadmill and gait-oriented exercise programs have also been shown to improve walking speed, stride length, and mobility performance in individuals with Parkinson’s disease [8]. Similarly, high-intensity exercise interventions have demonstrated beneficial effects on motor symptoms and functional performance [9]. The inclusion of aerobic conditioning, gait retraining, and task-oriented mobility exercises in the present study may therefore explain the observed improvements in mobility outcomes.

Significant improvement in balance performance, as reflected by increased Berg Balance Scale (BBS) scores, may be attributed to the incorporation of balance retraining, proprioceptive activities, and trunk stabilization exercises within the rehabilitation protocol. Previous studies have reported that exercise-based rehabilitation approaches, including Pilates and hydrotherapy interventions, contribute to improvements in balance and postural control in populations with Parkinson’s disease [14,15]. The combination of static and dynamic balance exercises used in the present study likely contributed to enhanced postural stability and reduced balance impairment.

Resistance training interventions have previously demonstrated improvements in muscle strength and physical performance in individuals with Parkinson’s disease [10]. Long-term progressive resistance exercise has additionally been associated with sustained functional benefits and enhanced activity participation [11]. In the present study, strengthening exercises, transfer training, and functional task practice may have contributed to improved functional performance.

Task-specific training and cueing strategies incorporated into the intervention may have further enhanced functional outcomes. Cued task-specific training has been shown to improve performance during functional activities such as sit-to-stand tasks in Parkinson’s disease [12]. Similarly, adaptive full-body movement training has demonstrated beneficial effects on bradykinesia and coordination [13]. These findings support the role of repetitive, function-oriented movement practice in improving motor performance and functional abilities.

The observed improvements may be attributed to the comprehensive multi-component rehabilitation approach, which incorporated gait training, balance retraining, strengthening exercises, flexibility training, postural correction, trunk stabilization, coordination activities, transfer training, and task-oriented functional exercises tailored according to disease severity and individual functional capacity. The stage-based progression of exercises may have facilitated gradual adaptation to increasing functional demands while ensuring patient safety and participation. Repetitive task-specific practice and progressive exercise training are known to enhance motor learning, neuromuscular coordination, postural control, muscular strength, movement initiation, and functional movement efficiency through sensorimotor facilitation and neuroplastic adaptation. Additionally, the integration of mobility and balance-oriented activities may have contributed to improved gait performance, reduced postural instability, enhanced balance control, and better overall physical performance in individuals with chronic Parkinson’s disease.

The improvement observed in the modified Hoehn and Yahr staging suggests that structured rehabilitation may positively influence functional disability and motor performance associated with chronic Parkinson’s disease. Although pharmacological management remains central to treatment, physiotherapy interventions contribute significantly to preserving mobility, improving postural control, and maintaining independence [7]. Exercise-induced neuroplastic adaptations and repetitive motor training may collectively support improved motor efficiency and functional performance [3].

The use of validated clinical outcome measures, including TUG, BBS, and the modified Hoehn and Yahr scale, strengthens the reliability and clinical relevance of the present findings. These assessment tools have demonstrated good validity, reliability, and responsiveness in Parkinson’s disease rehabilitation research [5,17-20].

While previous investigations have often focused on isolated interventions or early-stage Parkinson’s disease populations, the present study implemented a structured, stage-informed, ability-based rehabilitation approach tailored according to functional performance and patient tolerance. This individualized strategy may have contributed to the improvements observed across multiple functional domains.

Despite these positive findings, certain limitations should be acknowledged. The study included a relatively small sample size and utilized a single-group pretest-posttest quasi-experimental design without a control group, which may limit the generalizability of the findings and restrict the causal interpretation of the observed improvements. Additionally, potential confounding factors such as variations in disease severity, medication status, and individual functional capacity were not fully controlled and may have influenced the study outcomes. Further studies with larger sample sizes, control groups, and long-term follow-up are recommended to strengthen the evidence regarding the effectiveness of structured stage-based physiotherapy rehabilitation in individuals with chronic Parkinson’s disease.

In conclusion, the findings of the present study support the growing evidence that structured physiotherapy interventions can effectively improve mobility, balance, and functional performance in individuals with chronic Parkinson’s disease.

## Conclusions

The findings of the present study demonstrate that a structured stage-based physiotherapy rehabilitation program tailored according to disease severity can significantly improve functional mobility, balance, and overall functional performance in individuals with chronic Parkinson’s disease. The intervention also contributed to improvement in disease severity across different modified Hoehn and Yahr stages. These findings support the clinical value of progressive exercise-based rehabilitation as part of long-term management in chronic Parkinson’s disease. Further research involving larger sample sizes, controlled study designs, and long-term follow-up is recommended to establish the long-term effectiveness and generalizability of these findings.
